# Daily Energy Intake Distribution and Cognitive Performance in Non-Demented Individuals

**DOI:** 10.3390/nu15030673

**Published:** 2023-01-28

**Authors:** Dora Brikou, Sokratis Charisis, Archontoula Drouka, Stavroula Myrto Christodoulakou, Eva Ntanasi, Eirini Mamalaki, Vasilios C. Constadinides, Nikolaos Scarmeas, Mary Yannakoulia

**Affiliations:** 1 Department of Nutrition and Dietetics, Harokopio University, 17671 Athens, Greece; 2 Department of Neurology, University of Texas Health Science Center at San Antonio, San Antonio, TX 78229, USA; 3 1st Department of Neurology, Aiginition Hospital, Medical School, National and Kapodistrian University of Athens, 11528 Athens, Greece; 4 The Gertrude H. Sergievsky Center, Taub Institute for Research in Alzheimer’s Disease and the Aging Brain, Department of Neurology, Columbia University, New York, NY 10032, USA

**Keywords:** energy intake distribution, timing of food intake, dietary patterns, mild cognitive impairment, cognitive function, cognitive decline

## Abstract

Cognitive disorders have become important public health issues around the world. Studies evaluating the association between cognitive decline and food timing are lacking. The objective of this study was to examine the potential association between energy intake distribution during the day and cognitive performance in cognitively healthy and mildly cognitive impaired individuals. Data were derived from the ongoing Albion study which includes people aged 40 years or older who have a positive family history of cognitive disorder or concern about their cognitive status. A thorough dietary and cognitive assessment was performed. Participants consuming low energy intake at the beginning of the day or high energy at the end of the day had higher cognitive function compared to participants characterized by the opposite pattern. This trend remained statistically significant even after adjustment for potential confounders (*p* = 0.043). This study suggests that individuals with worse cognitive function may choose to eat earlier during the day, when cognitive performance is better, and it might be hypothesized that a meal pattern characterized by high energy consumption at the beginning of the day or low energy at the end of the day could be a marker of cognitive impairment.

## 1. Introduction

As life expectancy increases and the number of older people is growing, cognitive disorders have become an important rapidly growing public health problem around the world [1]. Currently, it is known that over 50 million people suffer from Alzheimer’s disease (AD) worldwide. AD is the most common type of dementia, and it is expected that this number will reach 81.1 million by 2040 and 152 million by 2050 [2]. These numbers imply a remarkable economic and social burden for not only healthcare systems, but also for families, caregivers and older people themselves [3]. Individuals who ultimately develop a degenerative dementia such as AD will likely pass through several stages of cognitive deterioration [4]. Mild cognitive impairment (MCI) has been defined as a transition state between healthy aging and AD [4]. Individuals who have MCI are at greater risk of developing AD compared to individuals with normal cognition [5]. More specifically, the rate of progression is variable but is in the range of 10% to 15% per year, in contrast to the progression rate from normal cognitive status to AD, which ranges from 1% to 2% per year [5]. Individuals with MCI are characterized by subjective memory impairment and objective memory impairment, compared to individuals of similar age and education, while their general cognitive function and the activities of daily living are still normal [4]. As pathological changes in the brain are initiated long before clinical manifestations [6], there is a large time period to implement prevention strategies that could potentially delay age-related cognitive decline and dementia.

However, there have been no effective medical therapies so far to prevent, delay, or modify dementia [7]. Therefore, other strategies should be considered. A substantial amount of evidence indicates that lifestyle factors such as physical activity and sleep habits could influence cognitive dysfunction [8,9]. Dietary intake is another lifestyle factor which is consistently proposed to exert beneficial or detrimental influences on cognition [10,11]. Particular nutrients, food groups, and dietary patterns have been linked to cognitive changes in older adults [10,12]. According to the GRADE approach there are seven key nutritional recommendations with regard to managing cognitive decline. Specifically, the significant consumption of mono- or poly- unsaturated fatty acids in combination with the low consumption of saturated fatty acids, vitamin D intake that is higher than the recommended daily allowance, high consumption of fruits and vegetables, as well as good adherence to a Mediterranean dietary pattern might protect against cognitive deterioration. Additionally, a ketogenic diet, low consumption of whole-fat dairy products or a caloric restriction are promising nutritional interventions, although the evidence does not yet support widespread uptake [12].

On the other hand, daily energy intake does not seem to be associated with cognitive function, as patients with AD and MCI do not differ from individuals with normal cognitive function in their total energy intake [13]. As can be observed, most of the existing studies have evaluated total dietary intake per day, and there has been much less focus on the timing of intake, i.e., the distribution of intake throughout the day and/or during specific eating occasions. The timing of food intake has been linked thus far with several health outcomes, including obesity and glycemic control. For example, higher caloric intake early compared to later in the day is associated with reduced susceptibility to weight gain [14] and greater weight loss [15], whereas the consumption of meals early in the day improves glycemic response [16]. Meal timing is considered to be an external signal which might interfere with the circadian clocks and may disrupt the physiologic harmony between predicted and actual behavior [14]. Such a desynchronization may favor the development of a wide range of disease-related processes, including obesity and its comorbidities [14].

In relation to cognitive decline, the evidence is scarce. It has been found that meal patterns oriented towards the early day, such as breakfast, compared to breakfast skipping [17], or having breakfast 4–6 times a week, compared to ≤3 times a week [18], are associated with decreased odds of having mild cognitive impairment. On the other hand, having lunch after 12:00 pm compared to having lunch earlier is associated with decreased odds of having mild cognitive impairment [18], whereas dinner consumption has not been associated with cognitive decline [17]. It is worthy of note that existing data refer to the consumption of the socially accepted main meals as classified by the individuals themselves, and there is little or no information on the specific time of day they were consumed or the energy content for each eating episode, whether meal or snack.

Considering that it is largely unknown whether the three-main-meal pattern (breakfast, lunch, and dinner) still exists [19], the consumption of main meals, in the aforementioned studies, is roughly self-assessed by relevant dietary behavior questionnaires and the fact that there is no consensus on meal and snack definition and classification [20], it seems more important, at a first stage, to explore and map energy intake distribution throughout the day. Data derived from 24 h dietary recalls containing information on the timing of each eating occasion allow for the evaluation of energy intake distribution ona continuum. Thus, the purpose of the present analysis is to examine the potential difference in energy intake distribution during the day between participants with normal cognitive function and mild cognitive impairment as well as the potential association between energy intake distribution during the day and cognitive performance in cognitive healthy and mildly cognitively impaired individuals.

## 2. Materials and Methods

### 2.1. Study Design and Population

ALBION (Aiginition Longitudinal Biomarker Investigation of Neurodegeneration) is a longitudinal study initiated in 2018. It takes place in the Cognitive Disorders Clinic of Aiginition Hospital of the National and Kapodistrian University of Athens, and is designed to address research questions regarding the preclinical and prodromal stages of AD. A detailed description of the study protocol has been published previously [21,22]. Briefly, study participants include people aged 40 years or older who are either referred by other specialists or self-referred to the cognitive disorders outpatient clinic of a tertiary university hospital. These participants may have a positive family history or concern about their cognitive status, or they may be asymptomatic with a commitment to contributing to medical science. Furthermore, in order to be included, a lumbar puncture as well as a whole-brain imaging on a 3T Philips Achieva-Tx MR scanner (Philips, Best, The Netherlands) should be performed. Exclusion criteria are diagnosis of dementia, neurological, psychiatric or medical conditions associated with a high risk of cognitive impairment or dementia, MRI contraindications, as well as the use of anticoagulant medication.

A thorough interview and a clinical examination were performed by specialist neurologists to assess all of the participants. Vital signs and physical strength data were also recorded. Participants’ weight and height were measured and Body Mass Index (weight/height^2^) was calculated. Each participant underwent an extensive assessment of several parameters, including several demographic (years of age, years of education, sex), medical, social, environmental, clinical, nutritional, neuropsychological determinants and lifestyle activities through a range of questionnaires. Furthermore, data from portable devices, neuroimaging techniques and biological samples were collected. Included individuals were diagnosed as either having normal cognitive function (NCF) or having at most mild cognitive deficits, i.e., mild cognitive impairment (MCI) as determined by a specialist neurologist after an extensive standardized neuropsychological assessment; diagnoses were reached using established diagnostic criteria [23]. An MCI diagnosis is assigned when the participant has cognitive complaints and a measurable deficit in cognition with a standard deviation below 1.5 in at least one domain in the absence of dementia or impairment in everyday functioning. The study protocol was approved by the National and Kapodistrian University Ethics Committee. Written informed consent was obtained from all participants at the time of enrollment.

### 2.2. Cognitive Function Assessment

Global cognition was assessed using the Mini Mental State Examination (MMSE) [24] and the Addenbrooke’s Cognitive Examination Revised (ACE-R) [25] by trained neuropsychologists. A variety of neuropsychological tests were performed to provide information on five main cognitive domains: (a) attention (Trail Making Test A [26] and Digit Span Forwards [27]), (b) executive function (Trail Making Test B [26], the Stroop Test [28], and Digit Span Backwards [27]), (c) visuo-spatial abilities (the Medical College of Georgia Complex Figure Test/copy and the visuo-spatial component of ACE-r), (d) memory (verbal memory: the Greek Verbal Learning Test and story recall, both immediate and delayed [29]; nonverbal memory: the Medical College of Georgia Complex Figure Test, both immediate and delayed), and (e) language (the semantic and phonological verbal fluency component of ACE-r, the language component of ACE-r, and a 40-item naming test). Participants’ raw scores on the individual neuropsychological tests for each cognitive domain were transformed to z-scores using mean and standard deviation values derived from the non-MCI group of the total study sample. Therefore, an average domain score for attention, executive and visual-spatial functioning, memory and language was produced. Individual cognitive domain scores were then averaged to calculate a global cognitive z score (a higher score indicated better performance).

### 2.3. Dietary Intake Assessment

Dietary intake was evaluated by four 24-h recalls using the five-step multiple-pass method [30], a method which can accurately assess energy and macronutrient intakes in both women and men [31,32]. Participants were asked by appropriately trained registered dietitians to report in detail all foods and beverages consumed the day before (i.e., between waking up in the morning and going to bed at night) the assessment. Specific timing as well as location, parallel activities, and companions were also recorded for each eating occasion. The first recall was conducted in person and the subsequent ones were conducted over the telephone. The telephone-administered recall was as effective as the face-to-face-administered recall [33]. Three of the recalls were conducted on weekdays and one on a weekend day in order to more accurately estimate usual intake throughout the week. Participants were not aware of the day of the recall in advance, so they could notchange their diet in anticipation of the interview. Energy and macronutrient intake were calculated per 2-h intervals using the dietary analysis software Nutritionist Pro^TM^ (version 4.2, 2007, Axxya Systems, WA, USA).

### 2.4. Statistical Analysis

Characteristics of participants with NCF were compared with those with MCI. For normally and non-normally distributed quantitative variables, a t-test and Mann–Whitney test were performed, respectively. For categorical data, Pearsons’*χ*^2^ test was used to check for differences between groups. The association between total energy intake and cognitive status as well as cognitive performance was also assessed using binary logistic and linear regression models, respectively. Models were adjusted for age, sex, education, and BMI.

We used generalized additive models (GAMs) to model the relationship between energy intake and time of day. A GAM is a generalized linear model with a linear predictor that includes smooth functions of one or more covariates, hence allowing the modeling of non-linear relationships [34]. Two different models were constructed:

(1) Energy intake trends for different levels of cognition

To assess for differential energy intake trends for different levels of cognition, we fitted a model of the following form:

g(μ_i_) = α_0_ + α_1_ Cognition_i_ + f(Time_i_) + f_c_(Time_i_ | Cognition = MCI)

μ_i_ ≡ E(Energy = Energy_i_ | Time = Time_i_),

Energy~Tw_p_ (μ, σ^2^) such as Var(Energy) = σ^2^μ^p^

where α_0_ is the model intercept (the mean energy of individuals in the reference [i.e., NCF] cognition category), α_1_ is the difference in mean energy between individuals with MCI and those with NCF, and f and f_c_ are centered smooth function of the time variable, representing the trend of energy over the course of the day for the reference cognition category and the deviation of the MCI cognition category from this energy trend, respectively. Energy follows a prespecified distribution family, and g is a monotonic and differentiable linearizing link function that transforms the expectation of the response variable (Energy) at a specific time point to the linear predictor. The models also included terms for random intercepts, considering that different participants may have different energy intakes at the beginning of the day, as well as random slopes, considering that different participants may have different energy intake trends over the course of the day (for simplicity, the random part of the model is not included in the above equation). Thin-plate regression splines were used to parametrize the f and f_c_ [35]. Considering the positively skewed distribution of energy intake data, different combinations of conditional distributions and link functions were tested; a Tweedie distribution family (Tw) with a log link function provided the best fit for the data. Model diagnostics revealed significant overdispersion and heteroscedasticity of deviance residuals for the default Gaussian family models, which was to be expected given the zero-inflated, positively skewed nature of energy intake data (Supplementary Figure S1). These issues were largely resolved by specifying a Tweedie distribution family (Tw) with a log link function (Supplementary Figure S2). Despite the significant improvement in model fit, a minor trend was still present in model residuals. This was likely related to some remaining temporal autocorrelation in model errors given the time series nature of our data (Supplementary Figure S3), and could have easily been addressed by including anautoregressive term in the model. However, the gam function of mgcv does not currently allow for autoregressive terms, and other functions that do, do not support the extended family of distributions, such as Tweedie, that gam supports [36].

The basisdimension (k) was set to 12 to allow for maximal flexibility (“wiggliness”), since GAMs remove redundant degrees of freedom, thus protecting from overfitting, by applying a “wiggliness” penalty equal to: λ$\int {\left[f\prime \prime \right]}^{2}\mathrm{dx}$, where λ is known as the smoothing parameter that controls the tradeoff between model fit and model smoothness [34]. Restricted estimated maximum likelihood (REML) was used to estimate λ through a Bayesian approach, since it has demonstrated better overall performance and numerical stability compared to generalized cross validation (model default) [37,38].

(2) Interaction between energy trends and global cognitive score

To assess for potential interaction between energy trends over the course of the day and global cognitive z-score, we applied the usual notion of statistical interaction to smooth functions, using the tensor product approach described by Simon Wood [34]. The fitted model for the interaction between energy trends and global cognitive score had the following form:

g(μ_i_) = α_0_ + f_1_(Time_i_) + f_2_(ZCO_i_) + f_3_(Time_i_,ZCO_i_)

Model parametrization was otherwise performed as previously described. Models were adjusted for age, sex, education, and BMI; the interaction terms of these variables with time were also included in the models to adjust for potential differential trends based on age, sex, education and BMI. Analyses were performed using R (R Core Team, 2021).

## 3. Results

A total of 104 participants were included in the analysis; 73 (70.2%) had NCF and 31 (29.8%) had MCI. The characteristics of the participants are presented in Table 1. Participants had a mean age of 65 ± 9 years and 13 ± 4 years of education; 65.5% were women. Total daily energy intake was 1829 ± 530 kcal; 42 ± 9% of energy derived from carbohydrates, 44 ± 7% from lipids, and 15 ± 3% from proteins. Participants with NCF had more years of education compared to those with MCI, *p* = 0.019. BMI, age and sex distribution did not differ between the two groups. Total daily energy intake as well as total daily intake of carbohydrates, proteins and lipids as percentage of energy intake did not differ between individuals with NCF and MCI. Participants with NCF consumed more grams of protein daily (*p* = 0.039) than those with MCI, whereas the daily consumption of carbohydrates and lipids in grams did not differ between the two groups. Similarly, participants with NCF and MCI did not differ in protein intake in terms of grams per kilogram of body weight. Moreover, total energy intake was not associated with cognitive status (*p* = 0.139, 95% CI: 0.998, 1.000) or cognitive function (*p* = 0.348, 95% CI: −85.850, 241.173).

**Figure 1:**
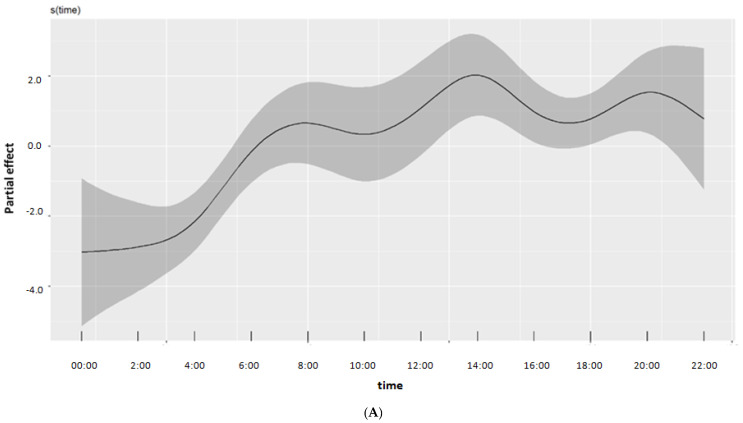

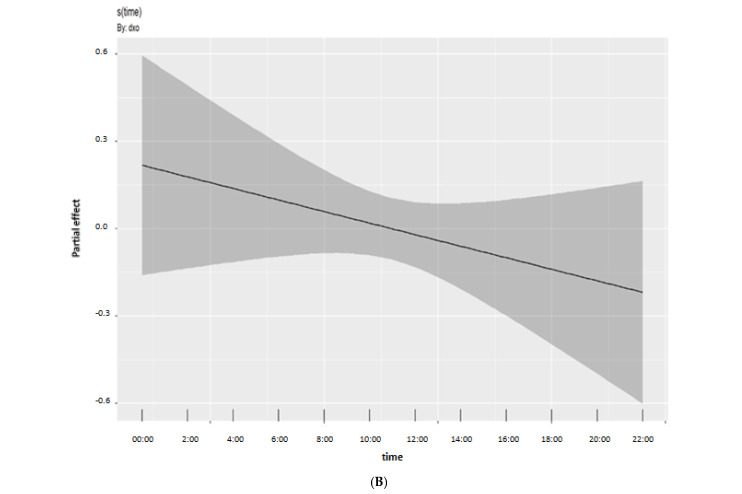
GAM−estimated smooth functions demonstrating the energy trend during the course of the day for the normal (NCF) cognition category (**A**), and the deviation of the MCI cognition category from this energy trend (**B**). Grey-shaded areas represent the respective 95% credible intervals. Energy values are plotted on the linear predictor (log) scale.

The model that was performed to evaluate the association between daily energy intake trends and different levels of cognition explained 43.3% of the variability for energy intake distribution during the day. Energy intake distribution did not differ between participants with NCF and MCI (Figure 1, Table 2). There was relatively higher uncertainty for estimates at the beginning and the end of the day (as is evident by the widened credible intervals) due to zero reported energy intake at these time points by the majority of study participants. We also observed differential energy intake trends during the day based on the participants’ sex. Differential energy intake trends during the day based on participants’ BMI were also detected.

**Figure 2:**
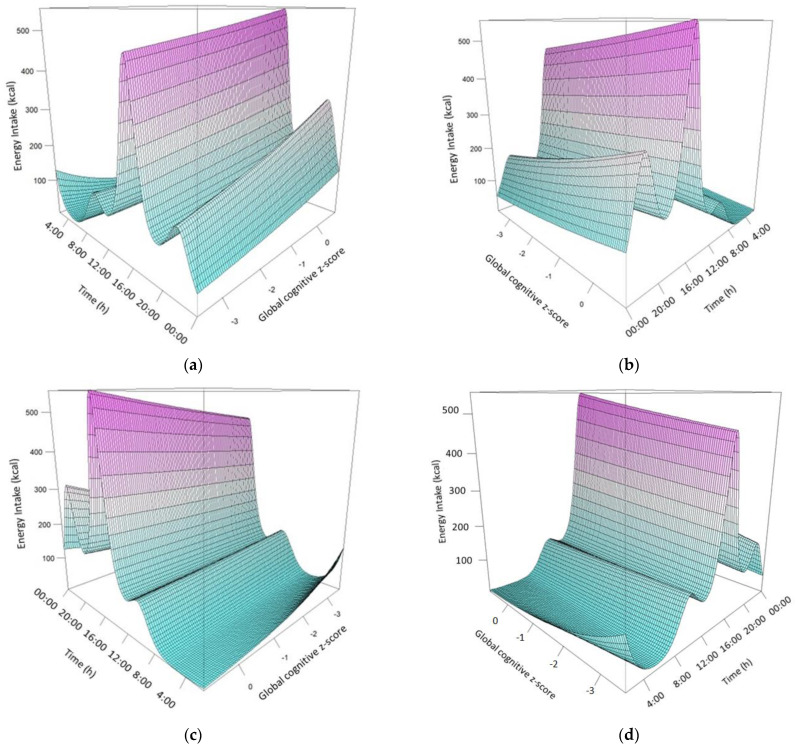
Multi−angle [(**a**): 45°, (**b**): 135°, (**c**): 225°, (**d**): 315°] Three-dimensional graphic illustration of daily energy distribution as a function of global cognitive z-score. The plot is derived from an adjusted GAM model with energy intake as the outcome and the tensor product of time with global cognitive z-score as the main predictor.

Furthermore, the model describing the association between daily energy intake trends and global cognitive z-score explained 44.3% of the variability for energy intake distribution during the day. Figure 2 presents the relation between energy intake distribution throughout the day and global cognitive z-score. Participants consuming low energy intake at the beginning of the day or high energy at the end of the day had higher cognitive function compared to participants characterized by the opposite pattern. This trend remained statistically significant (*p* =0.043) even after adjustment for potential confounders (sex, age, education, BMI), as well as for differential trends based on the aforementioned potential confounders (Table 3). This trend indicates that the energy intake patterns during the day might be related to the cognitive function of the individuals.

## 4. Discussion

The present cross-sectional study is the first study that evaluates associations between daily distribution of energy intake and cognition in adults aged 40 years or older. We examined the potential differential energy trends throughout the day for different levels of cognition (individuals with NCF vs. individuals with MCI) as well as the association between daily energy intake distribution and cognitive performance. We found that a pattern characterized by lower energy consumption at the beginning of the day and higher energy consumption at the end of the day is associated with better cognitive performance, whereas daily energy intake distribution did not differ between participants with NCF and MCI.

There are well established circadian rhythms in cognitive performance in humans, with worse performance in the early morning and late evening and the best performance somewhere in the middle of the daytime. Specifically, cognitive performance rises at 8:00 a.m., reaches a peak at about 4:00 p.m. and then begins to decline [39]. A circadian shift in intake patterns with the preponderance of calories consumed at breakfast and decreased energy consumption at dinner has been observed in seniors with AD and behavioral difficulties (mental disorganization and confusion) after 21 consecutive days of investigator-weighed food intake recording [40]. Furthermore, having lunch after 12:00 p.m. compared to having lunch earlier is associated with the decreased odds of having MCI [18]. Therefore, it seems that individuals with AD and MCI tend to prefer the early daytime for food intake. On the other hand, a tendency for meal sizes to increase over the day with peak intakes at noontime and early evening has been found in healthy young adults [41]. These findings are in agreement with our results indicating that consuming a significantenergy intake at the beginning of the day or low energy at the end of the day is associated with worse cognitive performance. Therefore, individuals with worse cognitive function may choose to eat earlier inthe day, when cognitive performance is better.

Regarding the potential underlying mechanisms, some hypotheses may be postulated. Older individuals sleep and wake earlier than younger ones and earlier relative to their nightly melatonin secretory episode [42]. Similarly, AD patients tend to go to bed earlier than healthy individuals of the same age range, and early in respect to their DLMO (dim light melatonin onset) clock time. This behavior may be due to their withdrawal from social and family activities [43],and it has beenobserved both in older people and in thosewith cognitive impairment. We found that participants with worse cognitive performance distribute their energy intake earlier; this change in eating habits with a tendency to reduce energy intake later in the day and increase energy intake early in the day could be the beginning of isolation from family and a marker of cognitive decline. On the other hand, going to bed earlier, with respect to DLMO clock time, has been reported to play a role in causing insomnia [44]. The discrepancy between bedtime and DLMO clock time could be a potential determinant of insomnia development [43], and thus this status could also influenceeating habits towards a preponderance of calories being consumed earlier rather than later in the day.

In addition, it has been found that healthy adults consume larger meals later in the day and that their satiety ratios decrease as the day progresses, indicating that humans develop less satiety from a given amount of food later in the day than earlier. This behavior is considered to happen spontaneously and represents eating in anticipation of the overnight fast [41]. In contrast to healthy adults, the acceptance of food is found to be the most likely at breakfast, while refusal of food occurs least often at this meal in demented individuals [45]. Moreover, hunger and satiety signals are likely disrupted secondary to neuronal degeneration [46,47], impacting pathways involved in food intake regulation. Therefore, individuals with worse cognitive function are characterized by low energy intake later in the day, perhapsbecause of the disruption of food intake regulation signals, leading to the loss of their spontaneous eating stimuli in order to be prepared for the following overnight fast. Taking the aforementioned into consideration, we may hypothesize that a meal pattern characterized by high energy consumption at the beginning of the day or low energy at the end of the day could be a marker of cognitive impairment.

Food timing has been found to play an important role in several disease-related entities. Higher caloric intake in the morning compared with later in the day has been linked with better health effects, such as greater weight loss [15] and improved glycemic response [16]. Breakfast consumption has also been associated with decreased odds of having mild cognitive impairment [17,18]. However, most studies so far have evaluated the energy intake of specific meals/time periods or meal frequency, and they have not examined energy intake distribution throughout the day. Furthermore, they are cross-sectional investigations and thus it is difficult to identify the causal relationships. Our hypothesis, as stated above, is that cognitive changes induce changes in eating patterns towards the early parts of the day, whereas in other health outcomes it is usually assumed that early eating predisposes to detrimental physiological or biochemical changes.

We detected an association only between energy intake distribution during the day and cognitive function, and no difference in daily energy patterns between individuals with NCF and MCI was observed. Cognitive function was assessed using the global cognitive z-score, which is a continuous variable, and better statistical power can be achieved as the study sample is not divided into specific groups.

Apart from the cross-sectional design of the study which did not allow us to determine causality, another limitation was the moderate sample size. However, the use of four 24-recalls along with the implemented statistical methods helped overcome potential power limitations by leveraging the multiple observations obtained during the course of the day. This method of dietary assessment allowed us to have analyses of time-specific food consumption and, by applying multiple recalls, for weekdays and weekends, we reduced the effects of random error (day-to-day variability in dietary intake) and ensured a true representation of energy intake. Another strength that should be noted is the detailed cognitive assessment through thorough clinical information as well as thevery extensive neuropsychological data that were collected. It should also be added that the ALBION study takes place in a specialist clinic of a tertiary university hospital.

## 5. Conclusions

This wasthe first study investigating the distribution of energy intake and cognitive function, indicating that higher energy consumption later in the day is associated with better cognitive performance. More studies are needed before generalizing our findings, and clinical trials are necessary to confirm the direction of the association. The fact that the disease process starts many years before the development of the disease symptoms [6] makes the early detection of cognitive impairment, through behavioral changes, a crucial matter in order to initiate effective interventions as early as possible. By increasing our knowledge on the role of timing of food intake in human health, new feasible strategies and recommendations can be developed.

## Figures and Tables

**Table 1:** Descriptive characteristics and eating patterns for the participants by cognitive status (*n* = 104).

Variables	ALL	NCF (N = 73)	MCI (N = 31)	*p*-Value
Sex (% female)	65.4	68.5	58.1	0.307
Age (years)	65± 9 (40, 79)	64± 9 (40, 79)	67± 7 (53, 79)	0.094
Education (years)	13 ± 4 (6, 22)	14 ± 4 (6, 22)	12 ± 4 (6, 17)	0.019
BMI (kg/m^2^)	27 ± 4 (15, 38)	27 ± 4 (15, 38)	27 ± 4 (21, 34)	0.962
Daily energy intake (kcal)	1829 ± 530 (878, 3555)	1889 ± 526 (993, 3554)	1688 ± 520 (878, 2856)	0.077
CHO				
g/day	189 ± 89 (76, 415)	194 ± 61 (76, 415)	178 ± 61 (92, 383)	0.237
% E	42 ± 9 (23, 70)	42 ± 9 (23,70)	43 ± 8 (24, 59)	0.508
Lipids				
g/day	89 ± 32 (35, 186)	93 ± 32 (35, 186)	81 ± 30 (36, 162)	0.071
% E	44 ± 7 (21, 59)	44 ± 8 (21, 59)	43 ± 6 (28, 54)	0.471
Proteins				
g/day	69 ± 23 (25, 143)	72 ± 23 (36, 143)	62 ± 23 (25, 132)	0.039
% E	15 ± 3 (9.5, 24)	15 ± 3 (10, 24)	15 ± 3 (9, 22)	0.275
g/kg body weight	0.94 ± 0.34 (0.35, 2.8)	0.98 ± 0.35 (0.46, 2.8)	0.85 ± 0.31 (0.35, 1.62)	0.053

Mean ± Standard deviation (minimum, maximum). Abbreviations: BMI = body mass index, NCF = normal cognitive function, MCI = mild cognitive function.

**Table 2:** Association between energy intake trends and different levels of cognition. Results from generalized additive models.

Parametric Terms			
	Estimate	Standard Error	*p*-Value
Intercept	4.21612	0.09798	<0.001
MCI	0.01036	0.07190	0.885
**Smooth Terms**			
	**Effective Degrees of Freedom**	**Reference Degrees of Freedom**	***p*-Value**
Time	9.720691	10.425	<0.001
Sex ^1^	5.854168	22.000	<0.001
Cognition ^2^	1.001234	1.002	0.242
Education	1.000490	1.001	0.066
Age	1.375659	1.672	0.807
BMI	1.000392	1.001	0.902
**Tensor Interaction Terms**			
Time, BMI	19.378845	27.967	0.024
Time, Age	1.002003	1.004	0.203
Time, Education	11.343028	16.509	0.185

Abbreviations: MCI = mild cognitive function, BMI = body mass index. ^1^ Male sex was specified as the reference sex category. ^2^ Normal cognitive function was specified as the reference cognition category.

**Table 3:** Association between energy intake trends and global cognitive score. Results from generalized additive models.

Parametric Terms			
	Estimate	Standard Error	*p*-Value
Intercept	4.19738	0.09464	<0.001
**Smooth terms**			
	**Effective Degrees of Freedom**	**Reference Degrees of Freedom**	***p*-Value**
Time	9.780887	10.499	<0.001
Sex ^1^	5.369277	22.000	<0.001
Education	1.000849	1.002	0.040
Age ^2^	1.003762	1.007	0.831
Global cognitive *z*-score	1.025011	1.049	0.262
BMI	1.000879	1.002	0.820
**Tensor Interaction Terms**			
Time, Global cognitive *z*-score	2.360638	2.958	0.043
Time, BMI	19.219090	27.799	0.025
Time, Age	1.002322	1.005	0.080
Time, Education	11.79925	17.091	0.152

Abbreviations: BMI = body mass index. ^1^ Male sex was specified as the reference sex category. ^2^ Normal cognitive function was specified as the reference cognition category.

## Data Availability

Not applicable.

## Supplementary Materials

The following supporting information can be downloaded at: https://www.mdpi.com/article/10.3390/nu15030673/s1, Figure S1: Kernel density plot for energy intake data; Figure S2: Deviance residual plots for: A. Gaussian distribution family generalized additive model with identity link function, B. Tweedie distribution family generalized additive model with log link function; Figure S3: Autocorrelation of model residuals.
