# Supplementary material for: Daily Energy Intake Distribution and Cognitive Performance in Non-Demented Individuals
Source: Nutrients. 2023 Jan 28;15(3):673. doi: 10.3390/nu15030673 (PMC9921864; doi:10.3390/nu15030673)
Supplement: Supplementary file 1 [file nutrients-15-00673-s001.zip › nutrients-2137668-supplementary.pdf]

Supplementary Materials:

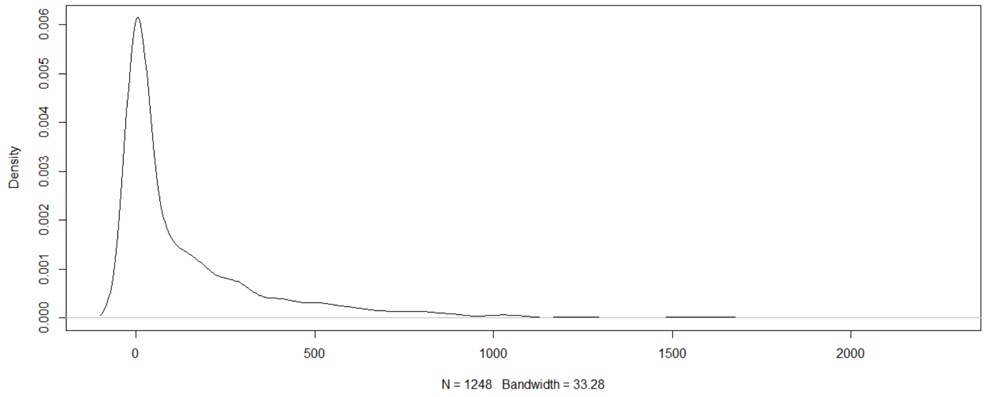

Supplementary Figure 1. Kernel density plot for energy intake data.

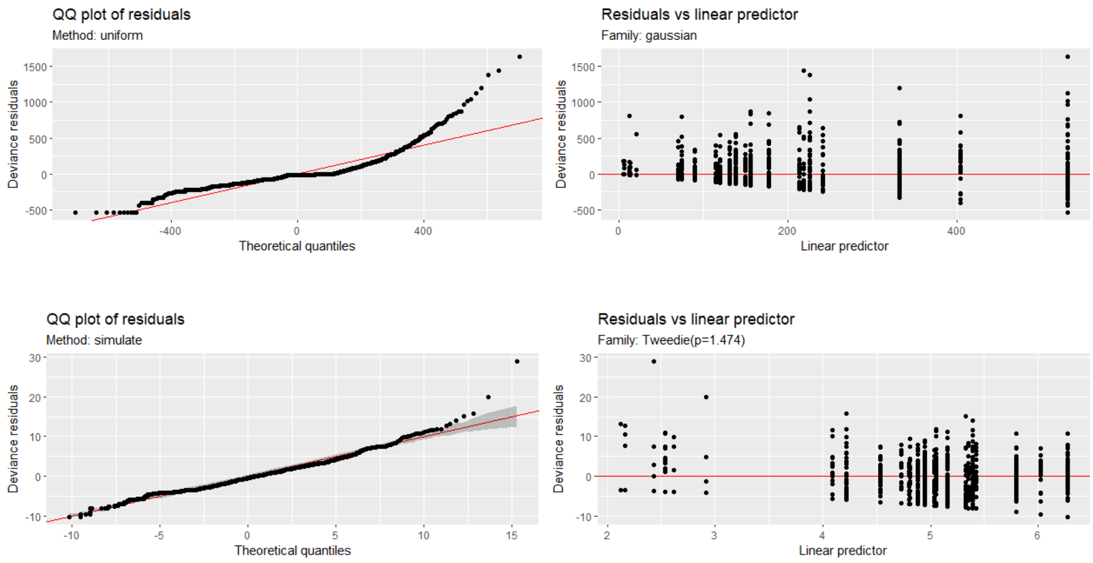

Supplementary Figure 2: Deviance residual plots for: A.Gaussian distribution family generalized additive model with identity link function, B. Tweedie distribution family generalized additive model with log link function.

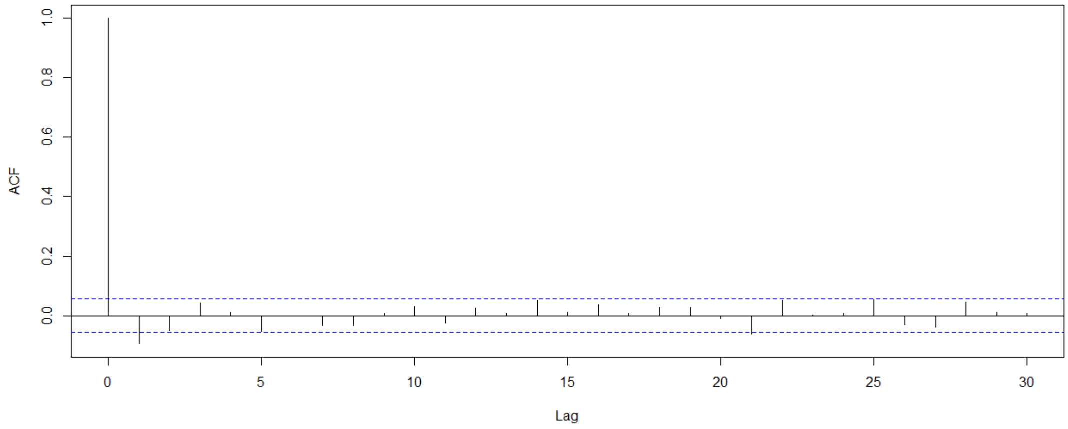

Supplementary Figure 3: Autocorrelation of model residuals.
